# Molecular mechanisms underlying electroconvulsive therapy-induced amnestic deficits: A decade of research

**DOI:** 10.4103/0019-5545.44745

**Published:** 2008

**Authors:** Chittaranjan Andrade

**Affiliations:** Department of Psychopharmacology, National Institute of Mental Health and Neurosciences, Bangalore-560 029, India

My dear colleagues and friends,

I am honored to have the privilege of delivering this oration before such an august gathering. I wish to pay my respects to the memory of the late Professor DLN Murthy Rao in whose name this oration was instituted. I thank my parents for their unwavering belief and support which nourished me during my formative years. I acknowledge with deep gratitude the influence of my teachers who, in more ways than just through education, were instrumental in shaping my growth. And, I place on record my indebtedness to the National Institute of Mental Health and Neurosciences, Bangalore, the institute which provided me with the opportunity to blossom in my career.

It is customary for the recipient of this award to showcase his research and to discuss its impact on the field. I am aware that I am best known for my work on electroconvulsive therapy (ECT) and psychopharmacology, especially in the context of animal models of investigation. However, listeners may be surprised and even amused to learn that, with the assistance of able students, colleagues, and collaborators at my institute and elsewhere, my fields of enquiry have been varied and wide. I am a curious person by nature and strongly believe that science knows no boundaries; if a question is worth asking, it is worth studying. As a result, in addition to ECT, I have worked and published in clinical psychopharmacology,[1–20] preclinical (basic science) psychopharmacology, [21–35] herbal medicine,[1,4,9,30,36–48] psychiatric phenomenology, [49–58] bipolar disorders,[8,11–13,56,59–65] statistics and research methodology, [66–76] family psychiatry[77–84] and fields such as environmental medicine, neuropsychology, education, and even snakebite.[85–92] My interest in these diverse areas persists to this very day. For those who may be curious, I have cited some representative references in this article; readers who would like a complete bibliography, or who desire reprints of specific articles, may write to me.

Without a shadow of doubt, an interest in ECT dominated my research during the past twenty-five years. Subjects that my colleagues and I addressed related to the history[93] and practice[94–96] of ECT; the efficacy[97–116] and adverse effects of the treatment in clinical and preclinical contexts;[117–126] methods and schedules of administration of ECT;[127–131] knowledge about, attitudes towards, and experiences with ECT;[132–142] and even literary issues.[143]

## FIELDS OF SPECIAL INTEREST

Four areas seized our especial interest: the use of herbal medicines in the attenuation of the cognitive adverse effects of ECT,[38–47] the mechanism of action of ECT,[144–151] electrical aspects of ECT[97,98,127,151–165] and the safety and efficacy of unmodified ECT;[166–172] we addressed these areas as independent as well as interacting entities. I summarize our findings in the passages that follow. For clarity in communication, I will use the generic term ‘ECT’ to refer to both electroconvulsive therapy (in humans) and electroconvulsive shocks (in animal models).

### Herbal medicines for the cognitive adverse effects of ECT

ECT induces anterograde and retrograde amnesia, as well as non-memory cognitive deficits. The amnestic deficits of ECT have been well characterized; whereas anterograde amnesia is mild and temporary, retrograde amnesia for autobiographical and nonautobiographical memories is frequent, and may be severe in a small proportion of patients; memories of lesser emotional salience are more vulnerable to loss. Although there is no evidence that ECT results in brain damage, and although there is evidence that ECT stimulates neuroplasticity and related changes in structures such as the hippocampus, amygdala, and prefrontal cortex, the use of electricity, the triggering of a seizure, and the cognitive adverse effects of the treatment are together responsible for the treatment being held in distrust.[134] To this extent, at least, the acceptability of ECT could improve should it prove feasible to contain the cognitive adverse effects without compromising the efficacy of the treatment.

Herbal medicines receive marketing approval in India without need for evidence of safety and efficacy.[46] We initiated a program to determine whether herbal treatments with putative neuroprotective effects could limit ECT-induced amnestic deficits. We used several different animal models of anterograde and retrograde amnesia in our experiments and obtained encouraging results with a complex herbal formulation,[38–42] with a simplified herbal formulation,[44–45] with the individual herbs Brahmi and Mandookaparni, separately and in combination,[47] but not with an aqueous extract of Shankapushpi.[43] Our findings have been summarized elsewhere along with a critical appraisal of the field, and a commentary on animal models and caveats concerning generalization from laboratory to clinical contexts.[46] Regrettably, as a result of a lack of funding, we were unable to do the translational research necessary to examine the safety and efficacy of herbal formulations in humans who are treated with ECT for psychiatric disorders; however, we did demonstrate that one formulation attenuates age-related cognitive decline in a community-based population.[1] The field remains open for further investigation.

### The mechanism of action of ECT

Today, so much is known about the mechanism of action of ECT that it would take a book to review the field. Our contribution to this area has been small. Using animal models, we showed that ECT downregulates dopamine autoreceptors and alpha-2 adrenoceptors, and upregulates dopamine postsynaptic receptors, thereby facilitating both dopaminergic and noradrenergic neurotransmission.[144–149,151] Importantly, for the first time in literature, we showed that maintenance ECT maintains the alpha-adrenergic receptor change[149] and also that high but not low dose electrical stimuli elicit the dopamine postsynaptic receptor upregulation that is seen with ECT.[151]

### Electrical aspects of ECT

Medications are dosed in terms of weight of the active ingredient. In ECT, the active ingredient is electricity. Curiously, most practitioners dose ECT in terms of the number of treatments administered; this is like asking patients to take a certain number of tablets without regard to the strength of the pill. Work by Sackeim and his colleagues, starting from the late 1980s, established that higher electrical doses with reference to the seizure threshold increase the likelihood of response in patients who receive unilateral ECT, and increase the speed of response whatever the electrode placement.[173–175] This encouraged us to probe deeper into electrical aspects of ECT.[97,98,127,151–165]

The stimulus in brief-pulse ECT is made up of four elements: pulse amplitude, pulse width, pulse frequency, and stimulus duration. These four elements can be combined in different ways to yield the same charge; that is, the same electrical dose. But, there is no assurance that differently constituted charges have identical biological effects.[155] In a tightly controlled study conducted in an animal model, we furnished unequivocal evidence that the seizure threshold is a variable point, and that, at constant electrical charge, it is lower when the electrical stimulus train duration is longer.[157] The importance of this study was underlined when the Association for Convulsive Therapy, USA, honored it with the ECT Investigator of the Year Award at its annual conference in San Francisco, during 2003.

In another study with far-reaching implications, we used power spectral electroencephalogram (EEG) analysis in an animal model to demonstrate how differently constituted electrical stimuli that were identical in charge could nevertheless differ in therapeutic efficacy and in adverse effect potential. We found that a narrow pulse width associated with high pulse frequency, especially in combination with a long stimulus train, were associated with three different EEG proxies of seizure efficacy: greater ictal power, greater postictal suppression, and greater interictal power, particularly in the lower frequency bands. In contrast, greater pulse width and lower pulse frequency showed no such associations. Stimulus-on time, number of pulses delivered, and rate of delivery of charge did not predict efficacy.[159] These findings could have much influence on the way ECT charges are constituted in clinical contexts. Clinical research on narrow pulse widths is now being pursued by several teams with encouraging results.

A chance observation during our work on the ECT seizure threshold led us to a dramatic new finding in neuroscience: that subconvulsive stimuli delivered to the brain lower the seizure threshold on subsequent days. In other words, the brain as a whole appears to respond more readily with a seizure discharge if previously primed with subconvulsive stimuli.[161] We confirmed this kindling effect in a controlled study.[162] Although we labeled the phenomenon ‘whole-brain kindling’, we later noted that what was actually kindled was probably only the brain territories involved in the triggering of the seizure.[163]

### Unmodified ECT

In a 1991 survey of the Indian Psychiatric Society, we found that only 44% of respondents to a postal questionnaire invariably administered modified ECT.[95] Our initial response was one of shock and indignation.[95,166] However, in an observational study,[167] we found that, notwithstanding the 20-40% incidence of musculoskeletal complications with unmodified ECT in historical Western studies, only 1 of 50 patients who received a course of 6 unmodified treatments actually experienced a spinal complication (which turned out to be subclinical). We subsequently revised our stance to the cautious position that modified ECT is desirable, but in exceptional circumstances unmodified ECT may be better than no ECT. We also examined issues related to ethics, and the definition of what might constitute exceptional circumstances for a country such as India.[168–171] In our most recent study, we confirmed our finding that musculoskeletal complications with unmodified ECT are rare in Indian patients, and suggested that benzodiazepine-modification of the seizure (resulting in drug-induced muscle relaxation) may be one of several explanations for our unexpected findings.[172]

## MECHANISMS UNDERLYING ECT-INDUCED AMNESTIC DEFICITS

Returning to the subject of our earlier research,[38–47] we reasoned that if we could understand what mechanisms explain ECT-induced amnestic deficits, we might be able to better develop treatments to diminish such deficits. Our lines of investigation followed two paths: we studied whether attenuating the systolic blood pressure surge during ECT results in decreased cognitive adverse effects[176–180] and we investigated the involvement of specific neurotransmitter mechanisms in ECT-induced amnestic deficits.[181–184] Curiously, two of the blood pressure surge studies themselves suggested neurotransmitter mechanisms.[177–178]

### Hypertensive surge and cognitive impairment with ECT

It has long been known that there is a sharp surge in systolic blood pressure during the ECT seizure.[180] Hypothetically, this surge may breach the blood-brain barrier, causing hyperperfusion-related mild cerebral edema as well as a leak of proteins and other macromolecules into the CSF and interstitial spaces in the brain. These changes may disturb neuronal functioning, predisposing to cognitive impairment.[179] We reviewed the clinical literature on the subject and found some support for this hypothesis.[176] Accordingly, we used an animal model to study whether the calcium channel blockers verapamil and felodipine, administered shortly before ECT, can reduce the amnestic deficits of the treatment. We chose both verapamil and felodipine because both belong to the same category of drug; and because the latter has only peripheral action.

In this study,[176] we demonstrated that just two once-daily ECT sufficed to induce retrograde amnesia in rats trained in the Hebb-Williams maze; verapamil or felodipine, administered half an hour before each ECT, attenuated this amnesia. On the surface, these finding support our hypothesis. However, we realized that other mechanisms could also have been be involved: cerebral vasodilatation, enhancement of cholinergic tone, and inhibition of calcium-mediated excitotoxic impairment of neuronal functioning.

In order to further investigate our hypothesis, we studied the effects of a different antihypertensive agent: sodium nitroprusside[177] and we separately asked whether *increasing* blood pressure, using phenylephrine, *worsens* amnestic deficits with ECT.[178]

We found that three once-daily ECT successfully induced retrograde amnesia in a passive avoidance task, and that the pre-ECT administration of nitroprusside, despite increasing the seizure duration, reduced the severity of this amnesia. Unexpectedly, nitroprusside also improved recall in control rats which did not receive ECT.

What do we make of these results? Nitroprusside may have attenuated the amnesia by reducing the systolic blood pressure surge during ECT. But, nitroprusside may also have worked by other mechanisms, such as by improving cerebral perfusion through vasodilatation. And, nitroprusside is a nitric oxide donor, and nitric oxide mechanisms are known to facilitate learning and memory. Most importantly, the improved recall in control rats suggested that nitroprusside could have nonspecific effects on cognition that are independent of ECT and the blood-brain barrier breach mechanism. As a result, these findings[177] again left us without a firm conclusion.

So, we moved to the phenylephrine study.[178] Phenylephrine is a nonselective alpha receptor agonist which can be expected to raise blood pressure. We examined whether the administration of phenylephrine immediately before ECT would increase ECT-induced amnesia. As before, we found that three once-daily ECT induced retrograde amnesia in a passive avoidance task. Surprisingly, (and even more so because we found that, like nitroprusside, it increased seizure duration), phenylephrine *attenuated* the ECT-induced amnestic deficits. An additional unexpected finding was that phenylephrine also improved recall in rats which received sham ECT. This led us to conclude that the adrenergic effects of phenylephrine nonspecifically improved memory functioning; in this context, adrenergic mechanisms have long been known to facilitate memory consolidation and storage. Since phenylephrine increases blood pressure, our most recent findings weakened the hypothesis that ECT-induced cognitive impairment is a consequence of cerebral edema and blood-brain barrier breach resulting from the intra-ECT hypertensive surge. However, it remained possible that the adrenergic cognitive-facilitatory mechanisms of phenylephrine could have more than counterbalanced the hypertensive surge cognition-disturbing mechanisms of ECT as augmented by the drug.

A limitation of these experiments is that we did not actually measure blood pressure changes with the drugs that we administered. Nevertheless, the findings that nitroprusside, phenylephrine, and calcium channel blockers attenuate ECT-induced amnestic deficits are still positive results worthy of clinical examination. As far as the hypertensive surge hypothesis is concerned, the field remains open for further study.

### Glucocorticoid mechanisms and ECT-induced amnesia

The hippocampus and the amygdala are both involved in learning and memory, and neurons in both structures richly express glucocorticoid receptors. Physiological activation of these receptors facilitates long-term potentiation (LTP) and neuroplasticity; that is, mechanisms which represent the cellular basis of learning and memory. However, excessive glucocorticoid stimulation (such as due to hypercortisolemia resulting from chronic stress, chronic depression, Cushing's syndrome, or the administration of exogenous steroids for therapeutic purposes) has been shown to induce a loss of dendritic spines and synapses, shrink the hippocampus, and impair cognition in both animal models and humans.[185]

Our review of literature[182] showed that ECT results in a hypercortisolemic surge that, though transient, lasts for hours after the seizure. Could this surge be responsible for the cognitive adverse effects of the treatment? Could blunting this surge attenuate ECT-induced cognitive impairments? We investigated the subject using the abortifacient drug mifepristone as an *in vivo* probe. In parentheses, mifepristone is used in gynecological practice because it is a competitive progesterone receptor antagonist; but mifepristone is also a highly potent glucocorticoid receptor antagonist. If ECT-induced hypercortisolemia stimulates hippocampal neurons physiologically, the transient hypercortisolemia could be a natural though incompletely effective defense against ECT-induced cognitive impairment; if so, mifepristone would worsen ECT-induced amnesia. But, if ECT-induced hypercortisolemia stimulates hippocampal neurons to a pathological degree, it would contribute to ECT-induced cognitive impairment; if so, mifepristone would reduce amnestic deficits with ECT.

**Figure 1:**
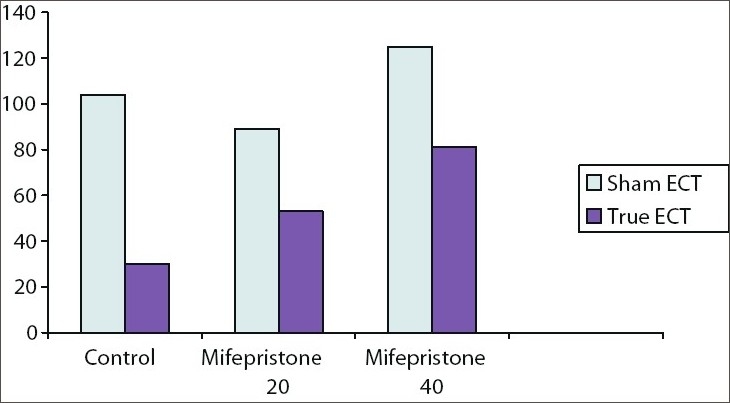
Mean recall scores in control and mifepristone (20mg/kg/day and 40 mg/kg/day) groups after five oncedaily ECT (lower scores indicate poorer recall; that is, greater retrograde amnesia). There was significant amnesia in the control but not mifepristone groups; recall scores were significantly higher in the mifepristone 40 mg/kg/day (but not 20 mg/kg/day) group relative to the control group

As we had done earlier, we investigated the matter in an animal model. We administered mifepristone in two different doses (20 mg/kg/day and 40 mg/kg/day) one hour before each of five once-daily ECTs to rats that had been trained in a passive avoidance task. We tested recall a day after the last ECT. A part of our results is presented in Figure 1. ECT resulted in retrograde amnesia in the control but not in the mifepristone groups. The higher dose of mifepristone was associated with significantly better recall scores relative to the control group. Thus, mifepristone clearly protected against ECT-induced retrograde amnesia. This implicates the ECT-induced hypercortisolemic surge (with consequent overstimulation of glucocorticoid receptors) as one mechanism of ECT-induced amnestic deficits, and suggests that glucocorticoid mechanisms can be modulated to protect against the development of these deficits.

### ECT and glutamateric excitotoxicity: A role for NMDA receptor, COX-2, and kynurenic acid modulation in ECT-induced amnesia

We next attempted something more ambitious: the study of the role of glutamatergic neurotransmission at the N-methyl D-aspartate (NMDA) receptor site, along with cyclooxygenase-2 (COX-2) and kynurenic acid lipid signalling mechanisms, in ECT-induced retrograde amnesia. Our work on this subject began with the well-known observation that chronic treatment with antiinflammatory drugs such as indomethacin in conditions such as rheumatoid arthritis is associated with a decreased risk of Alzheimer's disease; we therefore examined the neuroprotective effects of indomethacin and were pleasantly surprised to find that this drug reduced the severity of ECT-induced amnesia.[181] Indomethacin, however, is COX-nonselective and is, in fact, more effective against COX-1 than against COX-2; this is a negative for the drug because COX-2 is more important in learning mechanisms than COX-1.[183] Our review of literature[183] also showed that indomethacin increases kynurenic acid levels whereas COX-2 selective drugs such as celecoxib decrease kynurenic acid; as kynurenic acid dampens neurotransmission at all ionotropic glutamatergic receptor sites, and as glutamatergic neurotransmission is fundamental to LTP and neuroplasticity, celecoxib could be expected to demonstrate greater neuroprotective efficacy than indomethacin.

**Figure 2:**
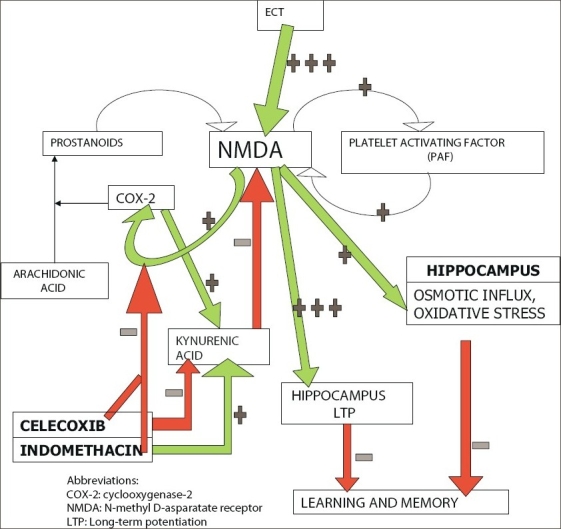
Glutamatergic mechanisms in learning, memory, and ECT-induced amnesia, and the europrotective mechanisms of indomethacin and celecoxib herein

Normally, glutamate released during learning stimulates the NMDA receptor, launching a cascade of downstream events [Figure 2]. These events include activation of COX-2 and thence a conversion of membrane arachidonic acid to endogenous prostanoids, as well as a conversion of endogenous cannabinoids (which dampen NMDA signalling) into novel prostaglandins (which increase NMDA activity). These events also include the activation of platelet activation factor (PAF). The COX-2 and PAF activation result in feedback augmentation of the NMDA signal. Other reverberating circuits (including those that involve retrograde neurotransmission through arachidonic acid) that amplify the NMDA signal also develop.[183,184] The net effect is glutamate- and NMDA-dependent initiation of LTP in the hippocampus, and thence the hardwiring of learning and memory through neuroplasticity changes.

Glutamatergic signaling is pathologically upregulated due to massive neuronal discharge during the ECT seizure. This may hypothetically result in a saturation of the cellular mechanisms that are responsible for hippocampal LTP and hence a decreased opportunity for the recruitment of these mechanisms for new learning. The consequence is ECT-induced amnesia.[183] Increased neuronal activity during the ECT seizure could additionally result in oxidative stress, and in an osmotic influx due to excessive (NMDA-mediated) entry of calcium into the cell; oxidative stress and osmotic load could both further contribute to ECT-induced cognitive impairment [Figure 2].

Under physiological conditions, COX-2 inhibitors inhibit learning and memory by dampening NMDA signaling. However, administration of COX-2 inhibitors pre-ECT could prevent glutamatergic excitotoxicity by blocking at least some of the reverberating circuits [Figure 2]; as a result, there could be less osmotic influx, less oxidative stress, and less saturation of LTP mechanisms. In consequence, ECT-induced amnesia may diminish. Based on the model that we constructed (simplified in Figure 2), we hypothesized that celecoxib would outperform indomethacin in this regard because, unlike indomethacin, celecoxib would decrease kynurenic acid, thereby facilitating normal NMDA signalling after the seizure.[183]

**Figure 3:**
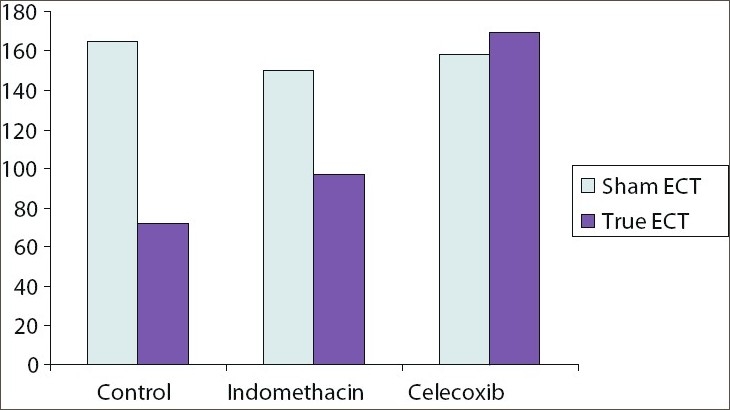
Mean recall scores in control, indomethacin (4 mg/kg/day), and celecoxib (15 mg/kg/day) groups after five once-daily ECT (lower scores indicate poorer recall; that is, greater retrograde amnesia). There was significant amnesia in the control but not indomethacin and celecoxib groups; recall scores were significantly higher with celecoxib (but not indomethacin) relative to controls.

We again conducted our studies in an animal model. We administered indomethacin (4 mg/kg/day) or celecoxib (15 mg/kg/day) to rats that received five once-daily ECT. We tested recall of passive avoidance learning a day after the last ECT. A part of our results is summarized in Figure 3. ECT resulted in retrograde amnesia in the control but not in the indomethacin and celecoxib groups. Whereas indomethacin partly protected against ECT-induced amnestic deficits, retrograde amnesia was completely prevented in the animals which received celecoxib. These findings support the model that we constructed [Figure 2] and suggest that glutamatergic and COX-2 mechanisms can be modulated to protect against ECT-induced retrograde amnesia in clinical contexts. In parentheses, interested readers would do well to read our studies in the original for a more detailed exposition of the mechanisms involved.

## WHERE DO WE GO FROM HERE?

As we described in an extensive review, we now know a great deal about the mechanisms underlying ECT-induced amnestic deficits[126] and there are extensive animal data to suggest pharmacological approaches towards the prevention of such deficits. Our own work suggests that certain herbal formulations, certain antihypertensive drugs (especially the calcium channel blockers), the glucocorticoid antagonist mifepristone, and certain antiinflammatory agents (especially the COX-2 inhibitors) justify further study in this regard. More specifically, translational research is now necessary to identify whether the findings from the laboratory can be extrapolated to the clinic.

### Concluding notes

This has been a long journey, but there are still miles and miles to go. I am conscious that I cannot alone do all that is left to do. I am conscious that there is a large pool of talent in this country that is looking for direction in researching important questions in psychiatry and psychopharmacology. I now wish to devote a greater proportion of my time to the development of research talent so that ambitious students and academicians in this country can feature more prominently on the global map. From my experience during the past few years, I have come to realize that possibly the only model which will succeed in this goal is one that is based on the academic institute. It would give me much pleasure to provide interested institutes in this country with training in research methods and statistics; to provide research questions and methodologies for the study of these questions; and to provide backup support for data analysis and report writing. If at the end of these exercises Indian psychiatry grows stronger in research capacity, my efforts would not have been in vain.
